# Dogme: a nextflow pipeline for reprocessing nanopore RNA and DNA modifications

**DOI:** 10.1093/bioinformatics/btag066

**Published:** 2026-03-02

**Authors:** Elnaz Abdollahzadeh, Ali Mortazavi

**Affiliations:** Department of Developmental and Cell Biology, UC Irvine, Irvine, CA 92697, United States; Center for Complex Biological Systems, UC Irvine, Irvine, CA 92697, United States

## Abstract

**Motivation:**

Oxford Nanopore (ONT) sequencing allows for the direct detection of RNA and DNA modifications from unamplified nucleic acids, which is a significant advantage over other platforms. However, the rapid updates to ONT basecalling models and the evolving landscape of computational tools for modification detection bring about challenges for reproducible and standardized analyses. To address these challenges, we developed Dogme to automate basecalling, alignment, modification detection, and transcript quantification. Dogme automates the reprocessing of ONT POD5 files by integrating basecalling using Dorado, read mapping using minimap2 and subsequent analysis steps such as running modkit. The pipeline supports three major types of sequencing data—direct RNA (dRNA), complementary DNA (cDNA), and genomic DNA (gDNA). Dogme facilitates detection of diverse RNA modifications supported by Dorado such as N6-methyladenosine (m6A), 5-methylcytosine (m5C), inosine, pseudouridine, 2’-O-methylation (Nm) and DNA methylation, while concurrently quantifying full-length transcript isoforms LR-Kallisto for transcript quantification for dRNA and cDNA.

**Results:**

We applied Dogme to three separate mouse C2C12 myoblast replicates using direct RNA sequencing on MinION flow cells. We detected 96 603 m6A, 43 476 m5C, 8829 inosine, 10 055 pseudouridine, and 30 320 Nm sites in three biological replicates. The pipeline produced reproducible modification profiles and transcript expression levels across replicates, demonstrating its utility for integrative long-read transcriptomic and epigenomic analyses.

**Availability and implementation:**

Dogme is implemented in Nextflow and is freely available under the MIT license at https://github.com/mortazavilab/dogme, with documentation provided for installation and usage.

## 1 Introduction

Long-read sequencing technologies have revolutionized genomics research by enabling the analysis of complex genomic loci (Logsdon *et al.* 2020) as well as full-length transcripts (Pardo-Palacios *et al*. 2024) that were previously difficult to characterize with short-read approaches alone. Oxford Nanopore Technologies (ONT) supports the direct detection of native RNA and DNA modifications on unamplified RNA (Leger *et al.* 2021) and DNA (Rand *et al.* 2017) molecules. Oxford Nanopore Technologies (ONT) stores the raw electrical signal generated by either the MinION or PromethION flowcells as each RNA or DNA molecule passes through a tiny nanopore into a POD5 file after a fixed number of reads or time, depending on user preferences. Thus, each sequencing run typically generates dozens to hundreds of POD5 files, which must be processed through several computational steps such as basecalling, mapping and modification calling to extract biologically meaningful information. While the Minknow user interface automates much of this for an initial run, not all basecalling features, including some of the newest modifications are available in a given release. Furthermore, ONT has been rapidly updating their Dorado basecalling software and models multiple times a year to achieve higher read quality, which can only be leveraged by reprocessing the raw POD5 data followed by repeating downstream analyses. This is a time-consuming process to repeat on a large number of samples that must be analyzed uniformly in large consortia such as ENCODE (Reese *et al.* 2023) and IGVF (IGVF Consortium 2024). To address the challenge of uniformly reprocessing samples generated across a long period of time such as the lifetime of a consortium, we developed Dogme, which is a Nextflow-based workflow that automates the processing of ONT raw signaling data through basecalling, alignment, modification detection, and transcript quantification. This workflow leverages the latest ONT basecalling models while ensuring computational reproducibility.

## 2 Methods

**Figure 1:**
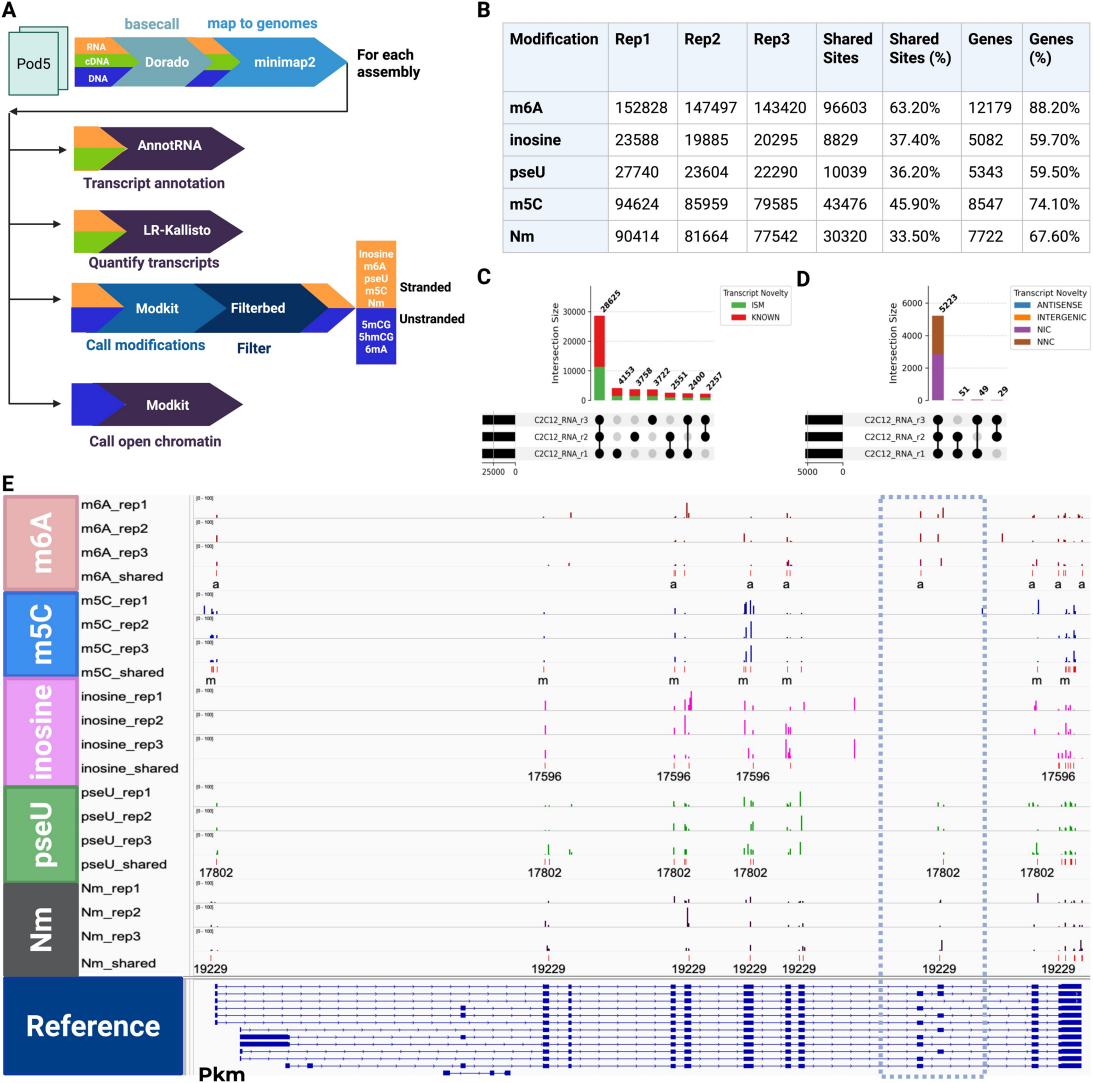
A uniform pipeline for reprocessing Nanopore data. (A) Dogme workflow. The pipeline processes dRNA, cDNA, and gDNA reads starting from basecalling (Dorado) and mapping (Minimap2). It then splits into parallel workflows for each assembly: transcript annotation (*AnnotRNA*), quantification (*LR-Kallisto*), and if appropriate, modification calling (*Modkit + Filterbed*) as well as open chromatin calling (*Modkit*). (B) Table of detected RNA modifications across three C2C12 myoblast replicates. Columns show site counts per replicate, sites shared across all three replicates, the number of expressed genes and the percentage of genes containing these modifications. (C) The intersection of detected “Known” and “Incomplete Splice Match” (ISM) transcript isoforms across the three biological replicates. (D) The intersection of shared novel transcript isoforms, classified as Antisense, Intergenic, Novel in Catalog (NIC), and Novel Not in Catalog (NNC). (E) RNA modification profiles for the *Pkm* gene. Tracks display the modification frequency for m6A, m5C, inosine, pseudouridine (pseU), and 2’-O-methylation (Nm) across replicates and shared sites. Dashed box shows alternatively spliced exons that have reproducible sites in one exon or the other.

Dogme is implemented in Nextflow, which enables scalable and reproducible scientific workflows across multiple computing environments (Di Tommaso *et al.* 2017). Dogme is specifically designed to handle three distinct types of sequencing data: direct RNA (dRNA) for detecting native RNA modifications, complementary DNA (cDNA) for high-accuracy transcript quantification without modifications, and genomic DNA (gDNA) for epigenetic analysis. All of the information to run Dogme is stored in a single configuration file, which specifies the sample type, the location of the starting POD5 files and all of the output folders, the modifications to be analyzed as well as the reference genomes and transcriptomes. The config file also has a section that specifies cluster-specific settings and queues. A separate file allows users to specify the paths of the pre-installed copies of Dorado (https://github.com/nanoporetech/dorado), Samtools (Li *et al.* 2009), Minimap2 (Li 2018), Modkit (https://github.com/nanoporetech/modkit), and Kallisto-bustools (Loving *et al.* 2025). The default Dogme workflow (Fig. 1A) starts by downloading the latest Dorado models, then proceeds with base-calling individual POD5 files using Dorado in parallel to produce one BAM file per POD5, which are then merged into a single unmapped BAM file stored in the bams folder. It is worth noting that Dorado is a GPU-intensive program, whereas all downstream steps are CPU-heavy. When used to analyze DNA or RNA modifications, this unmapped “modBAM” holds both the sequence information and modification probability scores for each nucleotide position. For dRNA and cDNA, a Fastq file is extracted from the BAM, which is then used to run long-read kallisto and bustools to quantify gene expression using a precomputed kmer index.

For all three sample types, reads are aligned to the genome using Minimap2. For dRNA and cDNA, Minimap2 uses the splice junctions from the gene annotation GTF file as a guide. Following Minimap2 alignment, the pipeline uses Modkit to extract RNA and DNA modifications from the aligned BAM files. Modkit leverages basecalling probabilities and positional information to identify modified bases either by sampling reads or using a user-defined threshold specified in the Dogme configuration file. Dogme finally applies an adjustable custom filtering step with thresholds of at least 3 supporting reads and a minimum modification frequency of 5% to minimize false positives while maintaining sensitivity. Importantly, Dogme is compatible with both stranded and unstranded data and supports analysis of both the plus and minus strands for RNA modifications, ensuring comprehensive coverage of transcriptomic and epigenetic features across all orientations.

Following alignment, dRNA and cDNA mapped BAMs are annotated with transcript information using the annotateRNA.py module. This step classifies reads according to the reference annotation and generates QC summary CSVs as well as TALON-compatible output files, facilitating downstream isoform discovery and quantification analyses. For genomic DNA (gDNA), the pipeline extends beyond methylation calling to infer open chromatin accessibility when labeled with exogenous m6A. Leveraging Modkit (v0.5+), Dogme calculates pileups of modification signals to produce open chromatin calls, outputting consolidated per-genome BED files and BedGraph tracks useful for identifying accessible regulatory regions.

Dogme also includes a reporting module (generate_report.py) that aggregates metrics across all processing steps. This generates an inventory_report.tsv and a qc_summary.csv, which track read counts, mapping statistics, and software versions used for every sample in the run.

Dogme provides multiple entry points into different parts of the pipeline. While the default workflow processes samples from raw signal (POD5) to final quantification, users can now trigger specific sub-workflows—such as basecall, remap, modkit, annotateRNA, or reports—allowing for the re-analysis of mapped BAMs or the generation of QC reports without re-running computationally expensive basecalling steps. For example, it is possible to start from PacBio CDNA data in BAM format and run the pipeline starting from the remap entry point. A pre-built Docker image that bundles all dependencies, including Dorado, Samtools, Minimap2, Modkit, and Kallisto, is available.

While community-standard nf-core (Ewels *et al.* 2020) pipelines such as nf-core/nanoseq provide robust frameworks for general Nanopore analysis, Dogme leverages Modkit to detect a broad, predefined spectrum of five RNA modifications (m6A, m5C, inosine, pseU, and Nm) and 3 DNA modifications (5mC, 5hmC, and 6 mA). Dogme also integrates LR-Kallisto for known isoform quantification and its own annotRNA tool for transcript annotation of mRNA and cDNA. Last but not least, Dogme expands beyond transcriptomics to support open chromatin accessibility calling from genomic DNA (gDNA) within the same automated framework, providing a multi-modal capability often requiring disparate workflows in other ecosystems.

## 3 Results

We tested Dogme using bulk directRNA sequencing from three biological replicates of mouse C2C12 myoblasts from the ENCODE project, on separate ONT MinION R10.4.1 flow cells sequenced to an average depth of 3.5 million reads per sample (ENCODE accession: ENCSR160HKZ). Reads were mapped on GRCm39 and quantified using GENCODE v36 annotations. We ran Dorado 1.0 on a SLURM HPC cluster with separate queues for NVIDIA A100 GPUs for the Dorado tasks and CPUs for the remaining tasks. Dorado currently supports the detection of five RNA modifications: N6-methyladenosine (m6A), 5-methylcytosine (m5C), inosine (A-to-I), pseudouridine (Ψ), and 2’-O-methylation on any base (Nm). These five modifications play crucial roles in regulating gene expression, RNA stability, splicing, and translation, making them essential components of cellular processes and potential biomarkers for disease (Li *et al.* 2023). We identified robust modification profiles across the three biological replicates (Fig. 1B). On average, we detected 147 915 m6A sites, 86 723 m5C sites, 21 256 inosine sites, 24 545 pseudouridine (pseU) sites, and 83 207 2’-O-methylation (Nm) sites per sample. A significant proportion of these sites were conserved across all three replicates, including 96 603 shared m6A sites (63.2% of the average total) and 43 476 shared m5C sites. These shared modifications were found in a broad set of expressed genes, with m6A appearing in 12 179 genes (88.2% of detected genes) and m5C in 8547 genes (74.1%).

We evaluated the reproducibility of transcript detection across replicates (Fig. 1C and D). The majority of detected transcripts corresponded to known isoforms and Incomplete Splice Matches (ISM), which showed high concordance across all three replicates (Fig. 1C). Furthermore, the pipeline successfully identified and classified novel isoforms—including Novel in Catalog (NIC) and Novel Not in Catalog (NNC) transcripts—demonstrating the workflow’s capability to capture reproducible novel splicing events alongside standard annotations (Fig. 1D). Examination of the gene Pkm (pyruvate kinase, muscle) illustrated the pipeline’s ability to detect modification patterns with high reproducibility. Across the three replicates, we consistently identified 21–29 m6A sites, 26–31 m5C sites, 28–31 Nm sites, 27–39 Ψ sites, and 18–24 low-frequency inosine sites within this gene. 14 m6A, 23 m5C, 16 Nm, 21 Ψ, and 12 inosine sites were shared across all replicates in Pkm. Interestingly, some of the shared modifications are found in alternatively spliced exons (Fig. 1E). Analysis of the modifications using long reads could therefore be used to tie modification patterns to specific isoforms. These results demonstrate that the Dogme pipeline enables robust and reproducible detection of RNA modifications from direct RNA sequencing data, providing a foundation for investigating epitranscriptomic dynamics in diverse biological contexts.

## 4 Summary

The development and validation of the Dogme pipeline addresses the computational challenges of uniformly reprocessing RNA and DNA modifications to leverage the latest ONT models. Dogme is designed to ease reanalysis starting from rebasecalling if desired as ONT continues to refine their models to improve sensitivity as well as specificity. Our results from C2C12 myoblasts demonstrate that this approach can consistently identify and quantify diverse RNA modifications across biological replicates with high reproducibility. It is remarkable that we detect each modification in at least half of the expressed genes in a sample (Fig. 1B). Furthermore, the ability to detect these patterns reproducibly across replicates indicates that Dorado can recover biological signals that are not technical noise, a critical requirement for confident epigenomic and epitranscriptomic analysis. While Dogme is not designed to analyze RNA modifications on specific transcripts, there are clear patterns of modifications that are isoform specific in the C2C12 data that would be very interesting to follow up on. Future directions for the Dogme pipeline include expanding modification detection capabilities, integrating additional downstream analysis tools for functional interpretation, adding support for single cell long read cDNA, and adapting the workflow for comparative analysis across different tissues, conditions, or species.

## Data Availability

An archival copy of DOGME used in this work is available from Zenodo with the following DOI: 10.5281/zenodo.17704722.
